# Supplementary figures and images for: Synthesis of Phenylboronic Acid-Functionalized Magnetic Nanoparticles for Sensitive Soil Enzyme Assays
Source: Molecules. 2022 Oct 14;27(20):6883. doi: 10.3390/molecules27206883 (PMC9611590; doi:10.3390/molecules27206883)

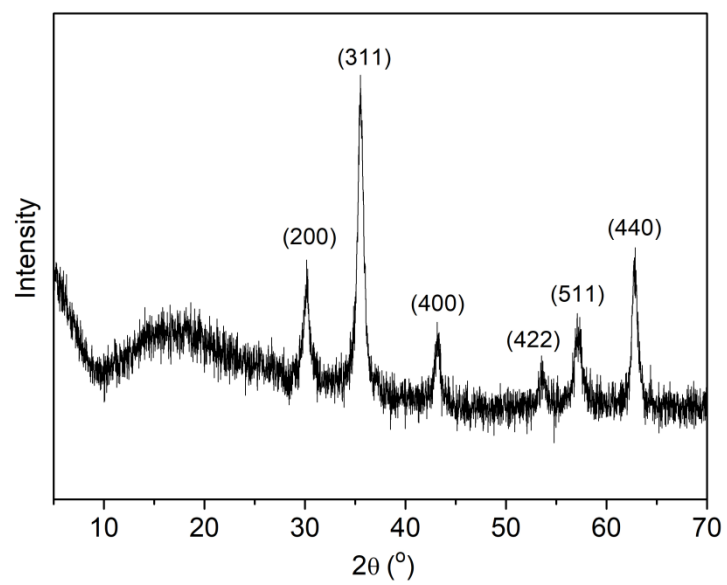

Figure S1. XRD pattern of the initial MNPs.

Supplement: Supplementary file 1 [file molecules-27-06883-s001.zip › molecules-1894224-supplementary.pdf]
